# SNP Analysis of Genes Implicated in T Cell Proliferation in Primary Biliary Cirrhosis

**DOI:** 10.1080/17402520500317859

**Published:** 2005-12

**Authors:** Sabine Oertelt, Thomas P. Kenny, Carlo Selmi, Pietro Invernizzi, Mauro Podda, M. Eric Gershwin

**Affiliations:** 1 Division of Rheumatology Allergy and Clinical Immunology University of California at Davis Davis CA 95616 USA; 2 Division of Internal Medicine Department of Medicine Surgery and Dentistry, San Paolo School of Medicine University of Milan Milan Italy

## Abstract

Previous studies on primary biliary cirrhosis (PBC) have focused on the role of T lymphocytes as potential effectors of tissue injury. We hypothesized that single nucleotide polymorphisms (SNPs) of genes involved in lymphocyte proliferation would be responsible for uncontrolled expansion of T cells and autoreactivity. To address this, we genotyped DNA from 154 patients with PBC and 166 ethnically matched healthy controls for SNPs of five candidate genes (60G/A CTLA-4, 1858 C/T LYP, -IVS9 C/T foxp3, p1323 C/G ICOS and -9606 T/C CD25) using a TaqMan assay.

We report herein a statistically significant decrease in homozygosity rate for the 60A⋆CTLA-4 allele in patients with PBC compared to controls (*p* = 0.0411). Moreover, we found a significant association of the same allele and of the LYP⋆T allele with anti-mitochondrial antibody (AMA) serum negativity (*p* = 0.0304 and 0.0094, respectively). No association between any of the other studied SNPs and PBC susceptibility, progression, or AMA status was observed. In conclusion, given the high prevalence of SNPs in CTLA-4 detected in numerous autoimmune diseases, we encourage a more detailed genetic analysis of this candidate gene. Further, although obtained from a limited number of AMA-negative subjects, our data suggest a potential genetic heterogeneity for this specific subgroup of patients with PBC.


*Clinical & Developmental Immunology*, December 2005; 12(4): 259–263

Taylor & Francis

Taylor & Francis Group

### SNP analysis of genes implicated in T cell proliferation in primary biliary cirrhosis

SABINE OERTELT[1,2], THOMAS P. KENNY[1], CARLO SELMI[1,2], PIETRO INVERNIZZI[2], MAURO PODDA[2], & M. ERIC GERSHWIN[1]

[1]*Division of Rheumatology, Allergy and Clinical Immunology, University of California at Davis, Davis, CA 95616, USA, and* [2]*Division of Internal Medicine, Department of Medicine, Surgery and Dentistry, San Paolo School of Medicine, University of Milan, Milan, Italy*

**Abstract**

Previous studies on primary biliary cirrhosis (PBC) have focused on the role of T lymphocytes as potential effectors of tissue injury. We hypothesized that single nucleotide polymorphisms (SNPs) of genes involved in lymphocyte proliferation would be responsible for uncontrolled expansion of T cells and autoreactivity. To address this, we genotyped DNA from 154 patients with PBC and 166 ethnically matched healthy controls for SNPs of five candidate genes (60G/A CTLA-4, 1858 C/T LYP, -IVS9 C/T foxp3, p1323 C/G ICOS and -9606 T/C CD25) using a TaqMan assay.

We report herein a statistically significant decrease in homozygosity rate for the 60A*CTLA-4 allele in patients with PBC compared to controls ($p = 0.0411$). Moreover, we found a significant association of the same allele and of the LYP*T allele with anti-mitochondrial antibody (AMA) serum negativity ($p = 0.0304$ and 0.0094, respectively). No association between any of the other studied SNPs and PBC susceptibility, progression, or AMA status was observed. In conclusion, given the high prevalence of SNPs in CTLA-4 detected in numerous autoimmune diseases, we encourage a more detailed genetic analysis of this candidate gene. Further, although obtained from a limited number of AMA-negative subjects, our data suggest a potential genetic heterogeneity for this specific subgroup of patients with PBC.

**Keywords:** *Primary biliary cirrhosis (PBC), single nucleotide polymorphism (SNP), CTLA-4, LYP, foxp3, ICOS*

### Introduction

Autoimmune diseases recognize a multifactorial etiopathogenesis (Roitt et al. 1992), most likely requiring environmental triggers to operate on a susceptible genetic background. In recent years intensive work has been performed to elucidate such genetic predisposition to autoimmune syndromes, especially following the completion of the human genome project. Based on the fundamental role of the major histocompatibility complex (MHC) in antigen presentation, much effort has been put into the dissection of its subtypes identifying significant and consistent associations only in some cases (Shiina et al. 2004). In this context, primary biliary cirrhosis (PBC), an autoimmune disease characterized by high-titer serum anti-mitochondrial autoantibodies (AMA) and tissue inflammation localized to the small and medium-size intrahepatic bile ducts (Kaplan 1996; Dienes et al. 1997), is interesting because it has no definite HLA association (Jones et al. 2003). The study of autoepitope localization (Van de Water et al. 1995), T lymphocyte reactivity (Shimoda et al. 1995; Kita et al. 2002), and local tissue reactivity (Yasoshima et al. 1995; Katayanagi et al. 1998) have suggested that the essential mechanism leading to organ damage is mediated by T cells (Ludwig et al. 1978; Hashimoto et al. 1993). We hypothesized that, similar to what observed in type I diabetes (Pop et al. 2005), autoimmune thyroid diseases (Setoguchi et al. 2005),

Correspondence: M. E. Gershwin, Division of Rheumatology, Allergy and Clinical Immunology, University of California at Davis School of Medicine, Genome and Biomedical Sciences Facility, 451 E Health Sciences Drive, suite 6510, Davis, CA 95616. Tel: 1 530 752 2884. Fax: 1 530 752 4669. E-mail: megershwin@ucdavis.edu

ISSN 1740-2522 print/ISSN 1740-2530 online © 2005 Taylor & Francis
DOI: 10.1080/17402520500317859

Table I. Primers used for SNP genotyping.

| Gene [SNP reference sequence] | Primers | Sequences |
|---|---|---|
| ICOS [rs 4270326] | ICOS-F | 5′-CAACAGGGAGACAATTCCTTCCCCC-3′ |
| | ICOS-R | 5′-AAGACATACCTACTAATTAAACTAA-3′ |
| Foxp3 [rs 2280883] | Foxp3- F | 5-GTCAATACACCCCCAACTGGGCACC-3 |
| | Foxp3-R | 5-TTCCCAACCTTTCCTTGTAACACCC-3′ |
| LYP [rs 2476601] | LYP-F | 5′-CCAGCTTCCTCAACCACAATAAATG-3′ |
| | LYP-R | 5′-CAACTGCTCCAAGGATAGATGATGA-3′ |
| CTLA4 [rs 3087243] | CTLA4-F | 5′-TCTTCACCACTATTTGGGATATAAC-3′ |
| | CTLA4-R | 5′-TGGGTTAACACAGACATAGCAGTCC-3′ |
| IL2RA/CD25 [rs 1570538] | IL2RA-F | 5′-CCTTAAAGACAGGATCTTGCTCTGT-3′ |
| | IL2RA-R | 5′-GAGTTCGAGGCTGCAGTCA-3′ |

and systemic lupus erythematosus (SLE) (Liu et al. 2004), a primary defect in T cell proliferation would explain the expansion of self-reactive clones in PBC. To investigate this phenomenon, we genotyped a large population of patients with PBC and healthy controls for the presence of single nucleotide polymorphisms (SNPs) of 5 genes involved in T cell expansion. In particular, we studied the prevalence of 60G/A CTLA-4 (cytotoxic T lymphocyte-associated antigen-4), 1858CT LYP (lymphoid thyrosine phosphatase) and -IVS9 C/T foxp3 SNPs, all previously associated with susceptibility to autoimmunity. Moreover, we studied the p1323 C/G ICOS (Inducible T cell costimulator) and -9606T/C CD25 SNPs (Table I).

### Materials and methods

### *Study population*

After written consent, blood samples from 154 patients with PBC consecutively enrolled at the Liver Clinic of the San Paolo School of Medicine (Milan, Italy) were obtained. Clinical characteristics of the enrolled population are presented in Table II. In all cases, the diagnosis of PBC was based on internationally accepted criteria: i.e. presence of serum AMA at titer higher than 1:40, histological evidence of liver damage compatible with PBC, and elevated alkaline phosphatase (>1.5X normal value) for longer than 6 months (Kaplan 1996). Disease stage was classified as early (histological stages I–II) or advanced (stages III–IV) PBC (Ludwig et al. 1978); the latter was also defined by a history of major complications related to portal hypertension. Seventy-six of 154 patients (49.4%) in our series presented early disease. Serum AMA testing was performed by indirect immunofluorescence. A population of 166 age and sex matched healthy subjects, sharing the same ethnical background as the patients, was used as control. The protocol corresponded to the guidelines of the Declaration of Helsinki (Edinburgh, 2000).

### *DNA extraction and SNP analysis*

DNA was extracted from whole blood using a commercially available kit (Instagene Matrix; Bio-Rad Laboratories, Segrate, Italy) and stored at −20°C until used for genotyping. SNP analysis was performed using TaqMan SNP Assay-on-Demand for ICOS and CTLA-4 and TaqMan Assay-by-Design for foxp3, LYP and CD25 (Applied Biosystems. Foster City, CA); primers were custom designed by Applied Biosystems (Table I). Polymerase chain reaction (PCR) amplifications were performed according to the manufacturer's instructions in 10 uL reactions, using TaqMan no AmpErase Universal Mastermix (Applied Biosystems), TaqMan primers and 5–10 ng of sample DNA. The assays were performed in 96 well PCR plates, each containing three negative and three genotyping controls. The PCR protocol included an initial denaturation step at 95°C for 10 min, 40 cycles of a 15 s 92°C denaturation

Table II. Demographic and clinical features of patients with PBC and controls enrolled in this study.

| | Patients with PBC | Control subjects |
|---|---|---|
| Number | 154 | 166 |
| Sex (F/M) | 142/12 | 160/6 |
| Age (years) | 66 ± 5 | 65 ± 8 |
| AMA positive | 132 (85.7%) | – |
| Advanced disease | 78 (50.6%) | – |
| Presence of ascites | 13 (8.4%) | – |
| Serum bilirubin (mg/dl) | 1.69 ± 3.379 | – |
| Prothrombin time (INR) | 1.04 ± 0.202 | – |
| Ongoing therapy with ursodeoxycholic acid (UDCA) | 130 (84.4%) | – |

Continuous variables are expressed as mean ± standard deviation.

Table III. Genotypes distribution in patients with PBC and healthy controls.

| | | | PBC ($n = 154$) | | | |
|---|---|---|---|---|---|---|
| | | Healthy controls ($n = 166$) | Total ($n$=154) | Early disease ($n = 76$) | Advanced disease ($n = 78$) | AMA negative ($n = 22$) |
| ICOS | GG | 5 (3.2%) | 3 (2.7%) | 0 | 3 (5%) | 0 |
| | GC | 47 (30.1%) | 26 (23.2%) | 11 (21.2%) | 15 (25%) | 3 (16.7%) |
| | CC | 104 (66.7%) | 83 (74.1%) | 41 (78.8%) | 42 (70%) | 15 (83.3%) |
| Foxp3 | GG | 48 (30.6%) | 44 (28.8%) | 18 (23.7%) | 26 (33.8%) | 6 (33.3%) |
| | AG | 69 (43.3%) | 68 (44.4%) | 34 (44.7%) | 34 (44.2%) | 5 (27.8%) |
| | AA | 43 (26.1%) | 41 (26.8%) | 24 (31.2%) | 17 (22.1%) | 7 (38.9%) |
| LYP | AA | 0 | 1 (0.7%) | 0 | 1 (1.4%) | 1 (5.6%) |
| | AG | 22 (13.3%) | 22 (15.2%) | 12 (16%) | 10 (14.3%) | 5 (27.8%) |
| | GG | 143 (86.7%) | 121 (83.4%) | 63 (84%) | 58 (82.9%) | 12 (66.7%) * |
| CTLA4 | GG | 49 (28.9%) | 40 (26%) | 21 (27.6%) | 19 (24.4%) | 4 (18.2%) |
| | AG | 72 (44.3%) | 87 (56.5%) | 43 (56.6%) | 44 (56.4%) | 16 (72.7%) |
| | AA | 45 (26.7%) | 27 (17.5%) ** | 12 (15.8%) | 15 (19.2%) | 2 (9.1%) *** |
| IL2RA/CD25 | AA | 39 (23.6%) | 24 (15.8%) | 13 (17.1%) | 11 (14.5%) | 3 (14.3%) |
| | AG | 76 (46.1%) | 74 (48.7%) | 35 (46.1%) | 39 (51.3%) | 9 (52.4%) |
| | GG | 51 (30.9%) | 54 (35.5%) | 28 (36.8%) | 26 (34.2%) | 7 (33.3%) |

Patients with PBC are also stratified according to disease stage and serum AMA status.

*$P = 0.0022$, $X^2 = 12.231$ AMA negative vs. healthy controls; **$P = 0.0411$, $X^2 = 6.384$ PBC vs. healthy controls. ***$P = 0.0304$, $X^2 = 6.984$ AMA negative vs. healthy controls.

followed by a 1 min 65°C annealing step. Allelic discrimination based on fluorescent quencher analysis (VIC–FAM) was assessed on a 7900HT Detection System (Applied Biosystems). Reproducibility of every set was confirmed by independent re-test of a randomly chosen subpopulation of samples.

### *Statistical analysis*

Differences in allelic frequencies were assessed by Fisher's exact test, followed by calculation of odd ratios and 95% confidence intervals if statistical significance was detected. Differences in genotype frequencies among the respective groups were evaluated by chi square calculation with one degree of freedom in 3 × 2 contingency tables. Stratification analysis was performed on the cohort to investigate the effects of sex, disease severity and antibody status. All analyses were two-tailed and $P$ values $< 0.05$ were considered as statistically significant. Calculations were performed using GraphPad Prism software, version 4.0 (GraphPad Software, Inc., San Diego, CA).

### *Results*

Allelic frequencies obtained in healthy controls in our study were consistent with previous data for 3 FoxP3, CTLA-4 and LYP SNPs while SNP frequencies for ICOS and CD25 were in accordance with GenBank frequencies. All SNP distributions were in Hardy-Weinberg equilibrium.

A statistically significant difference could be identified in the genotypic distribution of the 60G/A CTLA-4 SNP between patients and controls. Decreased homozygosity rate of the genomic A allele was detected in patients with PBC (17.5 vs. 26.7% in controls, $p = 0.0411$, Table III) while the GA and AA genotypes were accordingly associated with a decreased susceptibility to PBC (OR = 0.4966, confidence interval 0.2807–0.8784, $p = 0.0163$). A concomitant increase in heterozygosity (56.5% in PBC vs 44.3% in controls) appeared, associated with no significant modification in allelic distribution ($p = 0.4760$). A significant reproduction of the distribution was linked with AMA positivity ($p = 0.0304$). No correlation could be detected with disease severity.

No differential allelic expression among patients with PBC and healthy controls was identified for the 1858C/T LYP SNP, nor could any association with disease stage be evidenced. However, a correlation between LYP*T and serum AMA negativity was found (OR = 3.619, confidence interval: 1.431–9.153, $p = 0.0094$) and confirmed in genotypic distribution (Table III).

No discrepancies in total allelic frequencies or genotype distributions (Table III) among patients with PBC and controls and no divergence in relation to disease stage or AMA status was noted for -IVS9 C/T Foxp3, p1323 C/G ICOS and -9606 T/C CD25.

### Discussion

In the attempt to identify candidate genes potentially involved in the lymphocyte dysregulation characterizing PBC, we genotyped a large population of patients and controls and detected differences in genotype distribution for the 60G/A CTLA-4 SNP between patients and controls and for the CTLA-4 and LYP SNPs depending on the AMA status. Interestingly, CTLA-4 is a key inhibitor of uncontrolled T lymphocyte proliferation, fundamental for the maintenance of peripheral tolerance. Its transcript presents a vast number of SNPs, tied by variable degrees of linkage (Kristiansen et al. 2000). In particular, the 3′-UTR -60G/A SNP has been associated with susceptibility to SLE (Barreto et al. 2004; Torres et al. 2004), multiple sclerosis (Suppiah et al. 2005), and celiac disease (Hunt et al. 2005), either isolated or in association with linkage markers (Zhernakova et al. 2005). Moreover, Ueda et al. (2003) demonstrated that the association of the −60 A allele in linkage equilibrium with another genetic polymorphism, the CTLA-4* + 49A allele correlated with the production of the soluble (s) form of the receptor. S-CTLA-4 appears to have a direct regulatory function, binding in a competitive manner to the common ligands of CTLA-4 and CD28, B7-1 and B7-2, thus inhibiting proliferation by CD28 stimulation. Due to linkage patterns, the susceptibility allele G can be identified in healthy and affected individuals, making a complete linkage analysis of the region necessary for further elucidation. Encouraged by previous findings of CTLA-4 SNP association in PBC (Agarwal et al. 2000), we analyzed the -60G/A SNP confirming the pattern of celiac disease (Hunt et al. 2005), i.e. a decrease in homozygosity of the protective A allele, but we could not detect a concomitant increase in homozygosity of the putative susceptibility allele. The small subpopulation of AMA negative patients also resembles this genotype distribution. These apparently controversial results could be explained either by the limited size of the cohort or the previously described linkage pattern occurring in the region. Given the difficulty in recruiting extended cohorts of patients for rare diseases and ideally genetically homogeneous controls (Tsuneyama et al. 1998), a future analysis would have to focus on detailed sequencing and microsatellite dissection of the area, to allow a clarification of the genetic relevance of CTLA-4 to PBC. Issues correlated with cohort size also apply to the small subpopulation of patients with AMA-negative PBC, namely 5–10% of affected individuals (Miyakawa et al. 2001; Talwalkar et al. 2003). We are aware that the statistical power of the present comparisons and the obtained data might be questionable, mainly due to the difficulty in recruiting a sufficient number of subjects in this subgroup. Nonetheless, our findings on 1858T*LYP are intriguing, since this regulatory gene could be related to T to B lymphocyte interactions and autoantibody production. The polymorphic 1858T*LYP allele interferes with the binding of the negative regulatory kinase Csk to the activatory kinase LYP, thus deregulating the intracellular cascade and leading to lymphocyte expansion (Bottini et al. 2004). The polymorphism at position 1858 has been associated with autoimmune diseases (Hinks et al. 2005; Viken et al. 2005) but not to primary sclerosing cholangitis (PSC) (Viken et al. 2005), an autoimmune chronic cholestatic liver disease sharing several features with PBC but targeting also the large bile ducts (Wiesner et al. 1985). We could not identify a different distribution between PBC cases and healthy controls, but detected a significant association with AMA negativity.

Finally, neither an association with prevalence of PBC, nor correlation with disease severity or antibody status could be identified with any of the other analyzed SNPs. Considering the demonstrated expression of B7 by biliary epithelia (Tsuneyama et al. 1998), the potential role of ICOS, one of its natural ligands, warrants further investigation. Moreover, interesting data on the differential expression of T regulatory cells in autoimmune liver diseases (Longhi et al. 2004) drew our attention to CD25 and foxp3. Nonetheless, we could not identify any association with SNPs in both genes, thus preventing us from confirming a recent report on the expression of foxp3 in PBC (Park et al. 2005).

In conclusion, we have identified other possible candidate genes that might explain, at least in part, PBC onset and progression. The recent development of platforms allowing high-throughput analysis of a large number of SNPs at once (Syvanen 2005) may offer in the near future solid preliminary data to guide the selection of candidate polymorphisms for future studies on large series of patients and controls.

### References

Agarwal K, Jones DE, et al. 2000. CTLA-4 gene polymorphism confers susceptibility to primary biliary cirrhosis. J Hepatol 32:538–541.

Barreto M, Santos E, et al. 2004. Evidence for CTLA4 as a susceptibility gene for systemic lupus erythematosus. Eur J Hum Genet 12:620–626.

Bottini N, Musumeci L, et al. 2004. A functional variant of lymphoid tyrosine phosphatase is associated with type I diabetes. Nat Genet 36:337–338.

Dienes HP, Lohse AW, et al. 1997. Bile duct epithelia as target cells in primary biliary cirrhosis and primary sclerosing cholangitis. Virchows Arch 431:119–124.

Hashimoto E, Lindor KD, et al. 1993. Immunohistochemical characterization of hepatic lymphocytes in primary biliary cirrhosis in comparison with primary sclerosing cholangitis and autoimmune chronic active hepatitis. Mayo Clin Proc 68:1049–1055.

Hinks A, Barton A, et al. 2005. Association between the PTPN22 gene and rheumatoid arthritis and juvenile idiopathic arthritis in a UK population: Further support that PTPN22 is an autoimmunity gene. Arthritis Rheum 52:1694–1699.

Hunt KA, McGovern DP, et al. 2005. A common CTLA4 haplotype associated with coeliac disease. Eur J Hum Genet 13:440–444.

Jones DE, Donaldson PT. 2003. Genetic factors in the pathogenesis of primary biliary cirrhosis. Clin Liver Dis 7:841–864.

Kaplan MM. 1996. Primary biliary cirrhosis. N Engl J Med 335:1570–1580.

Katayanagi K, Kono N, et al. 1998. Isolation, culture and characterization of biliary epithelial cells from different anatomical levels of the intrahepatic and extrahepatic biliary tree from a mouse. Liver 18:90–98.

Kita H, Lian ZX, et al. 2002. Identification of HLA-A2-restricted CD8(+) cytotoxic T cell responses in primary biliary cirrhosis: T cell activation is augmented by immune complexes cross-presented by dendritic cells. J Exp Med 195:113–123.

Kristiansen OP, Larsen ZM, et al. 2000. CTLA-4 in autoimmune diseases—a general susceptibility gene to autoimmunity? Genes Immun 1:170–184.

Liu MF, Wang CR, et al. 2004. Decreased CD4+CD25+T cells in peripheral blood of patients with systemic lupus erythematosus. Scand J Immunol 59:198–202.

Longhi MS, Ma Y, et al. 2004. Impairment of CD4(+)CD25(+) regulatory T-cells in autoimmune liver disease. J Hepatol 41:31–37.

Ludwig J, Dickson ER, et al. 1978. staging of chronic nonsuppurative destructive cholangitis (syndrome of primary biliary cirrhosis). Virchows Arch A Pathol Anat Histol 379:103–112.

Miyakawa H, Tanaka A, et al. 2001. Detection of antimitochondrial autoantibodies in immunofluorescent AMA-negative patients with primary biliary cirrhosis using recombinant autoantigens. Hepatology 34:243–248.

Park O, Grishina I, et al. 2005. Analysis of the foxp3/Scurfin gene in Crohn's Disease. NY Acad Sci, 1051:218.

Pop SM, Wong CP, et al. 2005. Single cell analysis shows decreasing FoxP3 and TGFbeta1 coexpressing CD4+CD25+ regulatory T cells during autoimmune diabetes. J Exp Med 201:1333–1346.

Roitt IM, Hutchings PR, et al. 1992. The forces driving autoimmune disease. J Autoimmun 5:11–26.

Setoguchi R, Hori S, et al. 2005. Homeostatic maintenance of natural Foxp3(+) CD25(+) CD4(+) regulatory T cells by interleukin (IL)-2 and induction of autoimmune disease by IL-2 neutralization. J Exp Med 201:723–735.

Shiina T, Inok H, et al. 2004. An update of the HLA genomic region, locus information and disease associations: 2004. Tissue Antigens 64:631–649.

Shimoda S, Nakamura M, et al. 1995. HLA DRB4 0101-restricted immunodominant T cell autoepitope of pyruvate dehydrogenase complex in primary biliary cirrhosis: Evidence of molecular mimicry in human autoimmune diseases. J Exp Med 181: 1835–1845.

Suppiah V, Alloza I, et al. 2005. The CTLA4+49 A/G*G-CT60*G haplotype is associated with susceptibility to multiple sclerosis in Flanders. J Neuroimmunol 164:148–153.

Syvanen AC. 2005. Toward genome-wide SNP genotyping. Nat Genet 37:S5–10.

Talwalkar JA, Souto E, et al. 2003. Natural history of pruritus in primary biliary cirrhosis. Clin Gastroenterol Hepatol 1:297–302.

Torres B, Aguilar F, et al. 2004. Association of the CT60 marker of the CTLA4 gene with systemic lupus erythematosus. Arthritis Rheum 50:2211–2215.

Tsuneyama K, Harada K, et al. 1998. Expression of co-stimulatory factor B7-2 on the intrahepatic bile ducts in primary biliary cirrhosis and primary sclerosing cholangitis: An immunohistochemical study. J Pathol 186:126–130.

Ueda H, Howson JM, et al. 2003. Association of the T-cell regulatory gene CTLA4 with susceptibility to autoimmune disease. Nature 423:506–511.

Van de Water J, Ansari A, et al. 1995. Heterogeneity of autoreactive T cell clones specific for the E2 component of the pyruvate dehydrogenase complex in primary biliary cirrhosis. J Exp Med 181:723–733.

Viken MK, Amundsen SS, et al. 2005. Association analysis of the 1858C > T polymorphism in the PTPN22 gene in juvenile idiopathic arthritis and other autoimmune diseases. Genes Immun 6:271–273.

Wiesner RH, LaRusso NF, et al. 1985. Comparison of the clinicopathologic features of primary sclerosing cholangitis and primary biliary cirrhosis. Gastroenterology 88:108–114.

Yasoshima M, Nakanuma Y, et al. 1995. Immunohistochemical analysis of adhesion molecules in the micro-environment of portal tracts in relation to aberrant expression of PDC-E2 and HLA-DR on the bile ducts in primary biliary cirrhosis. J Pathol 175:319–325.

Zhernakova A, Eerligh P, et al. 2005. CTLA4 is differentially associated with autoimmune diseases in the Dutch population. Hum Genet :1–9.

